# Traditional Japanese Herbal Medicine Yokukansan Targets Distinct but Overlapping Mechanisms in Aged Mice and in the 5xFAD Mouse Model of Alzheimer’s Disease

**DOI:** 10.3389/fnagi.2018.00411

**Published:** 2018-12-17

**Authors:** Rahul Kaushik, Evgeny Morkovin, Jenny Schneeberg, Alessandro D. Confettura, Michael R. Kreutz, Oleg Senkov, Alexander Dityatev

**Affiliations:** ^1^Molecular Neuroplasticity, German Center for Neurodegenerative Diseases (DZNE), Magdeburg, Germany; ^2^Department of Fundamental Medicine and Biology, Volgograd State Medical University (VSMU), Volgograd, Russia; ^3^Laboratory of Genomic and Proteomic Research, Volgograd Medical Research Center (VMRC), Volgograd, Russia; ^4^Research Group Neuroplasticity, Leibniz Institute for Neurobiology (LG), Magdeburg, Germany; ^5^Leibniz Group “Dendritic Organelles and Synaptic Function”, University Medical Center Hamburg—Eppendorf, Center for Molecular Neurobiology (ZMNH), Hamburg, Germany; ^6^Center for Behavioral Brain Sciences (CBBS), Magdeburg, Germany; ^7^German Center for Neurodegenerative Diseases (DZNE), Magdeburg, Germany; ^8^Medical Faculty, Otto-von-Guericke University, Magdeburg, Germany

**Keywords:** yokukansan (TJ-54), aging, Alzheimer’s disease, microglia, astroglia, extracellular matrix, Aβ, herbal medicine

## Abstract

Yokukansan (YKS) is a traditional Japanese herbal medicine that has been used in humans for the treatment of several neurological conditions, such as age-related anxiety and behavioral and psychological symptoms (BPSD) related to multiple forms of dementia, including Alzheimer’s disease (AD). However, the cellular and molecular mechanisms targeted by YKS in the brain are not completely understood. Here, we compared the efficacy of YKS in ameliorating the age- and early-onset familial AD-related behavioral and cellular defects in two groups of animals: 18- to 22-month-old C57BL6/J wild-type mice and 6- to 9-month-old 5xFAD mice, as a transgenic mouse model of this form of AD. Animals were fed food pellets that contained YKS or vehicle. After 1–2 months of YKS treatment, we evaluated the cognitive improvements in both the aged and 5xFAD transgenic mice, and their brain tissues were further investigated to assess the molecular and cellular changes that occurred following YKS intake. Our results show that both the aged and 5xFAD mice exhibited impaired behavioral performance in novel object recognition and contextual fear conditioning (CFC) tasks, which was significantly improved by YKS. Further analyses of the brain tissue from these animals indicated that in aged mice, this improvement was associated with a reduction in astrogliosis, microglia activation and downregulation of the extracellular matrix (ECM), whereas in 5xFAD mice, none of these mechanisms were evident. These results show the differential action of YKS in healthy aged and 5xFAD mice. However, both aged and 5xFAD YKS-treated mice showed increased neuroprotective signaling through protein kinase B/Akt as the common mode of action. Our data suggest that YKS may impart its beneficial effects through Akt signaling in both 5xFAD mice and aged mice, with multiple additional mechanisms potentially contributing to its beneficial effects in aged animals.

## Introduction

Aging is a progressive process that is marked by complex and coherent changes. In humans, aging is a prime risk factor for many diseases, notably including the major forms of neurodegeneration. In particular, aging is a major determinant of the onset, progression, and pathogenesis of the most common form of dementia, Alzheimer’s disease (AD; Lindsay et al., 2002; Bertram and Tanzi, 2008). Interestingly, physiological aging and neurodegenerative diseases share many common symptoms, such as diminished cognitive abilities, neuronal loss, decreases in the number and efficacy of synapses, astrogliosis, microglial inflammation and an increase in neural extracellular matrix (ECM) proteins (Geula et al., 1998; Wiese et al., 2012; Végh et al., 2014a; Rodríguez-Arellano et al., 2016; Song and Dityatev, 2018). However, there are several crucial differences that underlie the differential brain function in aging and AD (Buckner, 2004), and their elucidation may be instrumental for designing effective drugs to ameliorate the symptoms or modify AD without major side effects. Present treatments that affect single molecular targets are not sufficiently effective or have a potentially high degree of associated risks (Cummings et al., 2014).

Natural herbal medicines that contain multiple biologically active substances have been in use for centuries and thus hold obvious therapeutic potential (Akhondzadeh et al., 2003; Cheung et al., 2015; Dey et al., 2017). One such herbal medicine is Yokukansan (YKS, referred to as *Yi*-*gan san* in China), which is a Kampo prescription that has been approved for human use by the Japanese Ministry of Health, Labor and Welfare for neurosis, insomnia, crying and irritability in children (Mizoguchi and Ikarashi, 2017a). YKS is prepared from seven herbaceous plants; it was developed in China in the 16th century and is traditionally used as a treatment for restlessness and agitation in children (Miyaoka et al., 2009). Multiple human studies have reported its benefits in age-related anxiety as well as in improving the behavioral and psychological symptoms (BPSD) associated with multiple forms of dementia, such as AD, dementia with Lewy bodies, Parkinson’s disease with dementia, frontotemporal dementia and vascular dementia (presented in more details in “Discussion” section). Animal studies suggest that YKS helps to not only alleviate BPSD-like symptoms (Egashira et al., 2008; Tabuchi et al., 2009) but also abrogate cognitive impairments (Ikarashi et al., 2009; Fujiwara et al., 2011).

The familial AD (5xFAD) mouse line is a widely studied mouse model of early familial AD that expresses three mutations (K670N/M671L, I716V and V717I) related to amyloid precursor protein (APP) and two mutations (M146L and L286V) related to Presenilin 1 (PS1) mutations under the control of Thy1 promoter (Oakley et al., 2006). 5xFAD mice have an increased Aβ load already at the age of 2 months, begin to develop plaques by the age of 3–4 months and show AD related synaptic and behavioral deficits and neuroinflammation by the age of 6 months, making them a suitable research model to investigate the early onset and progression of the disease (Oakley et al., 2006; Ohno et al., 2007; Eimer and Vassar, 2013; Bhattacharya et al., 2014).

Based on both *in vitro* and *in vivo* studies in various animal models—involving multiple brain areas, cell types and signaling molecules—several mechanisms have been suggested to explain the pharmacological action of YKS (Mizoguchi and Ikarashi, 2017a, b). However, a direct comparison of YKS-mediated effects in multiple models to dissect the common and model-specific underlying molecular and cellular mechanisms has not previously been accomplished. Thus, we aimed to systematically evaluate the ameliorating effects of YKS in aged and 5xFAD mice in terms of the activation of astrocytes and microglia, expression of the ECM, and neuronal survival signaling.

## Materials and Methods

### Animals

All animal experiments were conducted in accordance with ethical animal research standards defined by German law and the recommendations of the Ethical Committee on Animal Health and Care of the State of Saxony-Anhalt, Germany. The protocol 42502-2-1159 DZNE was approved by this committee. The present study used C57BL6/J male mice (hereafter referred to as aged mice, 18–22 months old at the beginning of the experiment) and 5xFAD male mice (hereafter referred to as 5xFAD mice, 6–9 months old, which were back-crossed on a C57BL6/J genetic background for at least 10 generations) divided into age-matched groups in accordance with YKS intake. The numbers of mice used were as follows: 10 aged mice treated with YKS, 9 aged controls, 7 5xFAD mice treated with YKS, and 7 5xFAD controls. At least 1 week prior to starting all procedures, mice were transferred from the breeding animal facility to the experimental animal facility, where they were individually housed with food and water available *ad libitum* on a reversed 12/12 light/dark cycle (light on at 9 P.M.). All behavioral experiments were performed during the dark phase of the cycle, i.e., when mice are active, under constant temperature and humidity.

### Yokukansan Preparation and Administration

YKS was prepared as a mixture of dried constituent plant extracts as previously described and shown in Table 1 (Ikarashi and Mizoguchi, 2016). The extracts were purchased from E-Fong Herbs manufactured by Yifang Pharmaceutical Corporation in China.

**Table 1:** The composition of Yokukansan (YKS).

Plant name	Weight of dried extract, g
Angelicae Radix (Dang Gui)	1.8
Atractylodes Lanceae Rhizoma (Fu Chuo Bai Zhu)	2.4
Bupleuri Radix (Chai Hui)	0.47
Poria (Fu Ling)	0.4
Glycyrrhizae Radix (Gan Cao)	0.5
Cnidii Rhizoma (Chuan Xiong)	1.3
Uncariae Radix (Gou Teng)	0.4
Total:	7.27

Briefly, the powdered extract was diluted in distilled water, and the resulting solution was added to homogenized food pellets (1% w/w of dry components). The paste was manually mixed and fitted into plastic tubes made of 3 ml syringes. Wet pellets containing YKS were pressed out and left at 50°C for 8 h to dry out.

During the food restriction procedure for the T-maze forced alternation task, the concentration of YKS was increased to compensate the reduced food intake. Control food pellets without YKS were prepared in the same way but without YKS to feed the control group of animals.

### Behavioral Analysis

All behavioral tests in the YKS-treated mice or their corresponding control mice were initiated after 1 month of feeding with the YKS-containing/control pellets, respectively. Animals were sequentially tested using the T-maze forced alternation task and novel object recognition task (NORT) followed by contextual fear conditioning (CFC) during the second month of feeding with YKS. Mice were sacrificed for further cellular and molecular analyses at the age of 20–24 months for the aged mice and 8–11 months for the 5xFAD mice.

### T-Maze Forced Alternation Task

The test of working memory was performed in an automated T-maze (O’Hara & Co., Ltd., Japan) as previously described (Shoji et al., 2012). Briefly, after food restriction (to 70% of normal consumption), we habituated the animals to the apparatus by placing them in the starting chamber for 10 min on two consecutive days. Each trial of the forced alternation task consisted of two runs: on the first (sample) run, the animals were forced to go into one arm of the T-maze to receive a food pellet (10 mg, colorless, odorless, round, AIN-76A, #1811213, TestDiet, St. Louis, MO, USA) as a reward; on the second (choice) run, both arms were open, but the reward was only available in the previously non-visited arm. During the first phase (learning), the animals underwent 10 trials per day without a delay between the sample and choice runs. When an animal’s performance reached a criteria of 80% correct choices per day, the 2nd phase (delayed alternation) was initiated with 0, 10, 30, 60 and 120 s delays between trials (10 trials for each interval in a pseudorandom order tested on five consecutive days).

### Novel Object Recognition Task

To further evaluate the effects of YKS on cognitive functions, we performed the NORT and CFC tests, as: (1) these are sensitive tests to detect changes in different cognitive domains; (2) related human tests exist, and the results could thus facilitate translational studies; and (3) aged and 5xFAD mice are impaired in both tests (Giannoni et al., 2013; Kesby et al., 2015; Dinkins et al., 2016; Larrayoz et al., 2017). A white plastic box (50 × 50 × 40 cm) was used for the NORT as described previously (Antunes and Biala, 2012). Briefly, the NORT consisted of three 10-min phases: the habituation phase followed by 24 h later familiarization phase, and 1 h later test phase. During the habituation phase, the animals were placed inside the box and left for 10 min to freely explore the area. During the familiarization phase, two similar objects were placed symmetrically at the center of the box. During the test phase, one of the objects was replaced by a novel object. The trial was performed twice to counterbalance the study with left or right object replacement. The exploration time in seconds for both objects was counted manually by a trained observer who was blind to the treatment conditions. The discrimination ratio (DR) was calculated as DR = 100 * [Exploration time (novel) − Exploration time (familiar)]/[Exploration time (novel) + Exploration time (familiar)].

### Contextual Fear Conditioning

CFC was performed as previously described (Minge et al., 2017). Briefly, we used a 20 × 20 × 30 cm Plexiglas chamber (Ugo Basil, Italy) with a chessboard-like pattern on the walls paired with foot shock, which served as the conditioned context (CC+), or plain gray walls and without a foot shock metal grid, which served as the non-aversive neutral context (CC−). Olfactory cues were also used: the chamber was precleaned with 75% ethanol or Meliseptol^®^ prior to the CC+ and CC− trials, respectively. During the training session, each mouse was placed into CC− and allowed to freely explore the chamber for 5 min. After 2 h, each mouse was placed into CC+ for 5 min. After 2 min of free exploration, the mouse received three foot shocks (0.5 mA, 1 s) delivered every 60 s. The retention sessions were performed 24 h (d1) and 72 h (d3) after the training session, during which the animals were placed into CC+ and CC− for 5 min with a 2 h interval without negative reinforcement. All sessions were recorded using a USB video camera (Microsoft, Redmond, WA, USA) and analyzed with video acquisition and analysis software (AnyMaze, USA) by a trained observer who was blind to the treatment conditions. The total time freezing was taken as a measure of fear-related memory and quantified as described previously (Senkov et al., 2006). The DR (D) between CC+ and CC− was calculated as *D* = 100 * (*F*_[CC+]_ − *F*_[CC−]_)/(*F*_[CC+]_ + *F*_[CC−]_), where *F*_[CC+]_ and *F*_[CC−]_ are the percent time spent freezing during the 5 min in CC+ and CC−, respectively.

### Immunohistochemistry

Hippocampal alterations, such as increased expression of ECM and activation of astrocytes and microglia, have been implicated in both aging and AD (Geula et al., 1998; Wiese et al., 2012; Végh et al., 2014b; Rodríguez-Arellano et al., 2016; Song and Dityatev, 2018). To investigate the effects of YKS on these cellular and molecular alterations, we performed histochemistry in hippocampal sections. Stainings were performed as previously described with modifications (Minge et al., 2017). Briefly, mice were sacrificed to isolate the hippocampi. One hippocampus was fixed with 4% paraformaldehyde (PFA) in phosphate-buffered saline (PBS) overnight at 4°C. The following day, the tissue was transferred to 30% sucrose in PBS for overnight cryoprotection, frozen in Tissue-Tek O.C.T. medium (Sakura Finetek USA Inc., Torrance, CA, USA) onto thin cork plates using liquid nitrogen and stored at −80°C until sectioning. Forty-micrometer thin sections were cut using a cryostat. Individual sections were collected into cryoprotection solution (one part ethylene glycol (Carl Roth, Cat no. 6881); one part glycerin (Carl Roth, Cat no. 3783) and two parts 1× PBS (Life Technologies, Carlsbad, CA, USA Cat no. 10010056)). The sections were washed three times with PBS for 10 min each wash at room temperature, followed by permeabilization with 0.5% Triton X-100 (Sigma) in phosphate buffer for 10 min at room temperature. The sections were subsequently blocked using a blocking buffer (0.4% Triton X-100 + 10% goat serum (Gibco; Cat no. 16210-064) + 0.1% glycine) in phosphate buffer for 45–60 min at room temperature. The sections were incubated with primary reagents, including biotinylated Wisteria Floribunda Agglutinin (WFA, 1:500, Sigma, Cat no. L1516), rabbit anti-aggrecan (1:200, Millipore, Cat no. AB1031), rabbit anti-ionized calcium-binding adaptor molecule 1 (anti-IBA1, 1:400, Wako, Cat no. 019-19741), mouse anti-glial fibrillary acidic protein (GFAP, 1:400, Sigma-Aldrich, Cat no. 3893) and mouse anti-human amyloid β (82E1), overnight at 4°C. The sections were washed three times with phosphate buffer and incubated with secondary reagents: Streptavidin 488 (1:1,000, Thermo Fisher Scientific, Waltham, MA, USA, Cat no. S11223), goat anti-rabbit Alexa Fluor 546 (1:1,000, Life Technologies, Carlsbad, CA, USA, Cat no. A11035), and goat anti-mouse Alexa Fluor 647 (1:1,000, Life Technologies, Carlsbad, CA, USA, Cat no. A21236). The sections were washed three times at RT for 10 min in phosphate buffer, incubated with DAPI (Invitrogen, Carlsbad, CA, USA Cat no. D1306; 1 mg/ml Hoechst 3342 in DMSO, diluted 1:400) and mounted using Fluoromount medium (Sigma; Cat no. F4680).

### Image Acquisition, Processing and Analysis

Images were acquired using a Zeiss confocal microscope (LSM 700) and EC Plan-Neofluar 20×/0.50 M27 objective by an experimenter blinded to the experimental groups. The hippocampal CA1 and CA3 areas were investigated for the cellular and molecular changes because of their strong involvement in the behavior paradigms used in the study and because the dysregulations of these hippocampal areas have been linked to AD (Padurariu et al., 2012). To measure the ECM expression and astroglial/microglial activation, we acquired images with defined properties of 16 bit, a frame of 512 × 512 pixels with the size of 1.25 × 1.25 μm^2^, in square areas that spanned CA1 or CA3 of the hippocampus. Open software ImageJ (Fiji) was used for image analysis.

### ECM Analysis

Biotinylated WFA and anti-aggrecan antibodies were used to label the ECM of perineuronal nets (PNNs). WFA labels the Gal- and GalNAc-terminated glycoepitopes on the core ECM proteins, such as lecticans, whereas the aggrecan antibody labels aggrecan core protein (Morawski et al., 2014; Matuszko et al., 2017). To count the WFA-positive (+) and aggrecan-immunopositive (Acan*+*) cells, 6–10 images were acquired per animal. Image analysis was performed by an experimenter blind to the experimental conditions. The area under analysis contained *stratum oriens* and *stratum pyramidale* from CA1 and CA3 that were manually defined (present on either side of CA2, which is defined by the rich presence of ECM staining). The numbers of Acan+ and WFA+ cells per area of CA1 and CA3 (in *stratum oriens* + *stratum pyramidale*) were counted manually.

### Astroglia Analysis

GFAP antibody recognizes glial fibrillary acidic protein and has been extensively used to investigate astrocytic activation (Nolte et al., 2001). To count GFAP+ cells, three images per animal were acquired and averaged. As the GFAP+ staining was uniform across the entire hippocampus under this magnification, we selected square regions of interest (374 × 374 μm^2^) in CA1 and CA3 areas. Autothresholding (moments dark option) and size exclusion criteria (size = 20) were applied in Fiji to count the number of GFAP-positive cells. Image analysis was performed by an experimenter blind to the experimental conditions.

### Microglia Analysis

Microglial inflammation has been linked to aging and AD (Davies et al., 2017; Martin et al., 2017). It is widely accepted that there are clear morphological changes in microglia after activation, such as enlargement of soma and a reduction of microglial processes (Glenn et al., 1992; Hovens et al., 2014). IBA1 antibody recognizes the microglia-specific protein called IBA1, which is commonly used as a microglia/macrophage-specific marker. A decrease in the arborization area and a corresponding increase in the somatic area are established indications of microglial activation (Hovens et al., 2014). For the evaluation of the arborization area, somatic area and number of IBA1+ cells, three images per animal were acquired and averaged. Image analysis was performed by an experimenter blind to the experimental conditions. Square regions of interest (374 × 374 μm^2^) were selected in the CA1 and CA3 areas. Two different thresholding and size exclusion criteria were applied to measure the total area covered by branching (Thresholding 1: autothresholding with mean dark option) and the soma area (Thresholding 2: autothresholding with triangle dark option) of IBA1+ cells. The soma area was subtracted from the total area to calculate the total area covered by IBA1 branching. Thresholding 2 with a size exclusion parameter enabled us to accurately count the number of IBA1+ cells. Differences in the averaged arborization area and somatic area per cell were calculated and averaged in each animal. In the 5xFAD animals, the microglial cells were clustered; thus, instead of a single cell analysis, we measured the total IBA1 intensity per mm^2^ of CA1 or CA3 (Supplementary Figure S3).

### Aβ Analysis

Hippocampal sections from YKS-treated and control animals were stained with Aβ antibodies that were raised against synthetic peptide for Human Amyloid (1–16) sequence and counterstained with 4′,6-diamidino-2-phenylindole (DAPI). We determined that the Aβ antibodies stained Aβ plaques with a strong DAPI signal in the center of the plaques. We subsequently used two different thresholds in Fiji to calculate the number of plaques (counted after thresholding of DAPI images) and the area covered by Aβ immunostaining. Supplementary Figure S4 indicates the reliability of the method applied, thus showing that the setting of two thresholds could be utilized to accurately measure the total plaque area (autothresholding with triangle option), and the DAPI signal could be used to count the number of Aβ plaques (autothresholding using intermodes option).

### Protein Sample Processing and Western Blotting

Since intake of YKS was oral, the active ingredients should affect the whole brain, including the hippocampus and cortex. This is noteworthy as CFC essentially depends on the hippocampal function, whereas performance in the NORT predominately depends on cortical areas, including the perirhinal cortex. Thus, we investigated several other aging and AD related mechanisms such as pAkt and insulin signaling in cortical tissue using Western blotting. Cortical tissue from control and YKS-treated aged and 5xFAD mice was extracted, instantly frozen and processed as follows.

### Protein Extraction

Snap-frozen cortices were homogenized in TBS buffer (0.25 M Tris-Base, 8% NaCl, pH 7.6) that contained protein inhibitor and phosphatase inhibitor cocktail (Roche). Proteins were extracted and solubilized by adding 1:1 SDS-buffer (8% SDS, 40% glycerol, 5% β-mercaptoethanol, 2 M Tris-HCl pH 6.8, 0.2% bromphenol blue) and denatured for 5 min at 95°C. The total protein concentration of the supernatant was measured using an amido black assay: protein samples were incubated with amido black solution (14.4 g of amido black in 1 ml of 9:1 methanol-acetic acid solution), centrifuged and washed three times with methanol-acetic acid solution. The air-dried pellet was dissolved in 100 mM NaOH solution, and the optic density was measured at 620 nm. Bovine serum albumin (BSA) was used as a standard.

### Western Blotting

Total protein extracts were separated in 5%–20% gradient acrylamide gel for SDS-PAGE, transferred to a nitrocellulose membrane and probed with specific antibodies against phospho-Akt (pAkt; phospho-Ser473, Cell Signaling, 1:1,000, Cat no. 4051, raised against synthetic phosphopeptide corresponding to residues around Ser473 of mouse Akt), Akt (40D4, Cell Signaling, 1:2,000, Cat no. 2920, detects the C-terminus of the protein), InsR (4B8, Cell Signaling, 1:1,000, Cat no. 3025, raised against synthetic peptide corresponding to residues surrounding Tyr999 of human insulin receptor β), IGF-1R (Cell Signaling, 1:1,000, Cat no. 3027, detects the C-terminus of the protein) and mouse anti-β-actin (Sigma-Aldrich Cat no. A5441), at 4°C for 12 h. The membranes were initially blocked with 5% nonfat milk or BSA in TBS-T (0.25 M Tris-Base, 8% NaCl, pH 7.6, 0.1% Tween 20). The membranes were washed four times for 10 min in TBS-T and incubated with secondary anti-mouse IgG-HRP (Dianova, 1:20,000, Cat no.111-035-146) or anti-rabbit IgG-HRP (Dianova, 1:20,000, Cat no. 111-035-144) antibodies for 1 h 30 min. The membranes were washed again and developed with an ECL system (Luminata Western HRP Substrate from Millipore or SuperSignal West Pico from Thermo Fisher Scientific, Waltham, MA, USA).

### Statistical Analysis

Statistical analysis was performed using GraphPad Prism 5.0 (GraphPad Software Inc., La Jolla, CA, USA) and Statistica 8.0 (StatSoft, Tulsa, OK, USA) software. All data are shown as the mean ± SEM. The hypothesis that experimental distributions follow the Gaussian law was verified using the Kolmogorov-Smirnov, Shapiro-Wilk, or D’Agostino tests. Pairwise comparisons were performed using Student’s *t*-test for Gaussian distributions; otherwise, the Mann-Whitney test was employed. Factorial analysis was performed using two-way analysis of variance (ANOVA) with the Bonferroni *post hoc* test.

## Results

To investigate the effects of YKS on the behavior, brain histology and biochemistry of aged and 5xFAD mice, half of the animals from both genotypes were fed either their normal food alone (referred to as control) or mixed with YKS (YKS-treated) in a randomized manner. The mice were subsequently sacrificed to isolate the tissue for the histochemical analysis of ECM expression, astrogliosis, microglial activation Aβ-plaque counting and biochemical analysis of various cell signaling mechanisms relevant to aging and AD.

### YKS Does Not Improve Working Memory in Aged and 5xFAD Mice

To test the effects of YKS on working memory, we used the T-maze forced alternation task. The learning curves and a number of days needed to reach the criteria of 80% correct choices per day were not affected by YKS in both groups of animals i.e., aged and 5xFAD mice. In aged animals, the YKS treatment had no effect on the number of correct choices either on phase 1 or phase 2 (*p* = 0.3090 and *p* = 0.8446, respectively, two-way ANOVA; Supplementary Figure S1). There was also no effect of delay interval in phase 2 (*p* = 0.0626), but the interaction between interval and treatment was significant (*p* = 0.0466, two-way ANOVA, Supplementary Figure S1). In 5xFAD mice, similar to aged mice, the total number of correct choices either on phase 1 or phase 2 was not affected by YKS (*p* = 0.2963 and *p* = 0.0501, respectively, two-way ANOVA). In contrast to aged mice, 5xFAD mice showed worse performance during delayed alternation phase, especially after 60 or 120 s delays between trials (*p* = 0.002 for the effect of delay interval, *p* = 0.7766 for interaction between interval and treatment, two-way ANOVA, Supplementary Figure S2).

### YKS Rescues Novel Object Recognition in the 5xFAD Mouse Model of Alzheimer’s Disease

**Figure 1:**
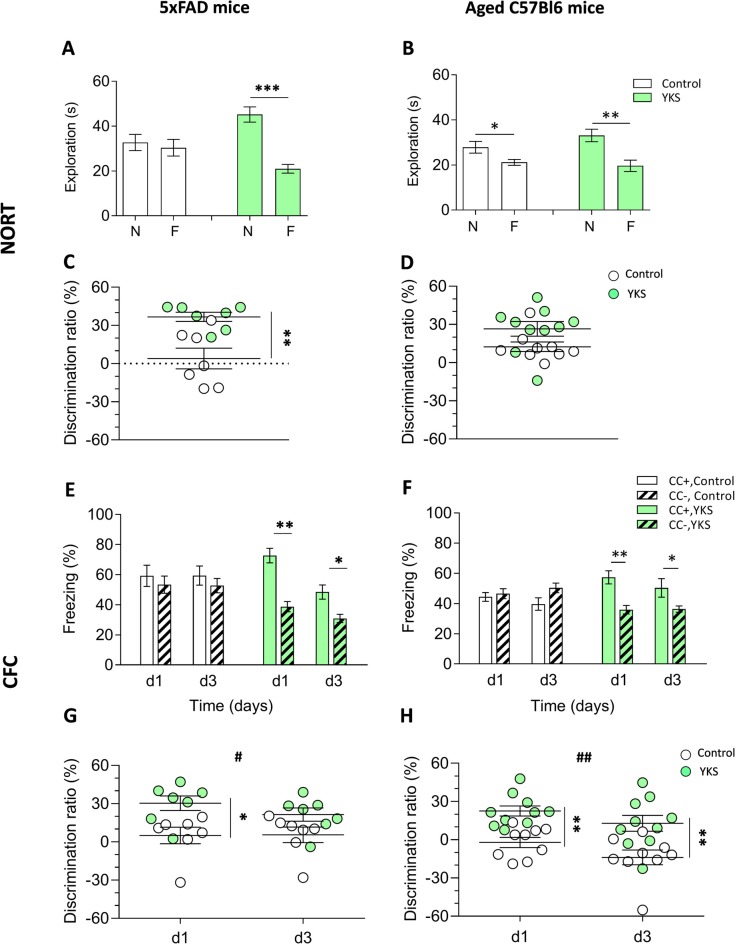
Effects of Yokukansan (YKS) on the behavior of 5xFAD and aged mice. Left: control and YKS-treated 5xFAD mice. Right: control and YKS-treated aged mice. **(A,D)** Novel object recognition task (NORT). The time spent exploring novel (N) and familiar (F) objects **(A,B)** and N/F discrimination ratio (DR; **C,D**) are shown. Exploration of each object was measured as the time intervals when the animal’s head faced toward the object and remained in the 2.5 cm area around the object. **(E,H)** Contextual fear conditioning (CFC) test. The percentage of time spent freezing **(E,F)** and CC+/CC− DR **(G,H)** on days 1 and 3 after fear conditioning are shown. Freezing was measured as time intervals when the animal remained motionless in a horizontal tense posture. The DRs were calculated for each animal as described in the“Materials and Methods” section. Bar graphs show the mean ± SEM values. *p < 0.05, **p < 0.01, ***p < 0.001 represent significant differences between treatment groups (within the same trial) and ^#^*p* < 0.05, ^##^*p* < 0.01 represent significant differences between different trials. *N* = 9 for control and *N* = 10 for YKS-treated aged mice and *N* = 7 for both control and YKS-treated 5xFAD mice.

To evaluate the potential effect of YKS on more long-term memory, we performed the NORT. 5xFAD mice fed with normal food showed no discrimination between the novel and familiar objects: the exploration time spent near these objects was 32.7 ± 3.6 s and 30.4 ± 3.7 s, respectively. However, the YKS group showed a significant improvement in the test, with 45.2 ± 3.4 s and 21.0 ± 1.9 s for the novel and familiar object exploration time, respectively. There was no difference (*p* = 0.66, paired *t*-test) in the time spent at the novel vs. familiar objects for the control group; however, a significant increase in the time spent at the novel object was identified for the YKS group (*p* = 0.0002, paired *t*-test; Figure 1A). Analysis of the DR also indicated a significant difference between the control and YKS groups (*p* = 0.0041, Mann-Whitney test; Figure 1C).

The aged mice fed with normal food showed a weak but significant discrimination between two objects (*p* = 0.018, paired *t*-test), with times spent at the novel and familiar objects of 27.86 ± 2.61 s and 21.17 ± 1.3 s, respectively (Figure 1B). The aged mice fed with YKS showed a high object discrimination (*p* = 0.0028, paired *t*-test), spending 33.11 ± 2.75 s vs. 19.65 ± 2.52 s at the novel and familiar objects, respectively. There was a strong tendency of YKS to improve the DR (*p* = 0.053, Mann-Whitney test; Figure 1D).

### YKS Rescues Context Discrimination in Aged and 5xFAD Mice

As shown in Figures 1E–H, YKS in the aged mice had a similar effect as in the 5xFAD mice, significantly improving discrimination between the conditioned (CC+) and neutral (CC−) contexts. In CC+, the control group froze 44.4 ± 2.9 s and 39.7 ± 4.2 s at days 1 and 3, respectively, while the YKS group froze 57.4 ± 4.4 s and 50.37 ± 6.1 s. The freezing time in CC− of the control group was similar to their times in CC+ on days 1 and 3: 46.6 ± 3.3 s and 50.4 ± 3.2 s; however, in the YKS group, the mice froze significantly less than in CC+: 35.9 ± 2.8 s and 36.3 ± 2.0 s, respectively. Two-way repeated measures (RM) ANOVA indicated a difference in the CC+ values only for the factor time (*p* = 0.04) and not for treatment with YKS (*p* = 0.06); however, for the CC− values, two-way RM ANOVA indicated a difference between the control and YKS groups (*p* = 0.0028), whereas time did not play a role (*p* = 0.27). The differences between CC+ and CC− were not significant on any testing day for the control group (*p* = 0.60 and *p* = 0.13, for days 1 and 3, respectively); however, for the YKS group, the difference was highly significant (*p* = 0.0011 and *p* = 0.023, for days 1 and 3, respectively). Analysis of the DR also showed a highly significant effect of YKS on CC+/CC− discrimination (*p* = 0.001, two-way RM ANOVA), and time also had an effect (*p* = 0.0029). Moreover, the Bonferroni *post hoc* test indicated a similar difference in discrimination between the two groups (*p* < 0.01) for both days. In 5xFAD mice, the effects of YKS appeared even more prominently. In the control group we found no difference between CC+ and CC− freezing times on day 1 (59.2 ± 7.0 s vs. 53.3 ± 5.7 s, *p* = 0.062) and day 3 (59.4 ± 6.3 s vs. 52.7 ± 4.8 s, *p* = 0.71). In the YKS-treated group, CC− freezing time on day 1 decreased in comparison to the control group (38.7 ± 3.4 s vs. 53.3 ± 5.7 s), while CC+ freezing time increased (72.7 ± 4.8 s vs. 59.2 ± 7.0 s), so they become significantly different (*p* = 0.002, paired *t*-test). On day 3 the difference between freezing times in CC+ and CC− in the YKS group was also significant (48.4 ± 4.7 vs. 30.9 ± 2.7 s, *p* = 0.0075). The DR was also affected by YKS treatment (*p* = 0.0175, two-way RM ANOVA). The values on day 1 were significantly higher in the YKS group than in controls (*p* < 0.05), but on day 3 no difference between groups was detected. The effect of time was not significant (*p* = 0.283, two-way RM ANOVA).

### YKS Decreases Expression of Aggrecan-Based ECM of Perineuronal Nets in Aged but Not in 5xFAD Mice

**Figure 2:**
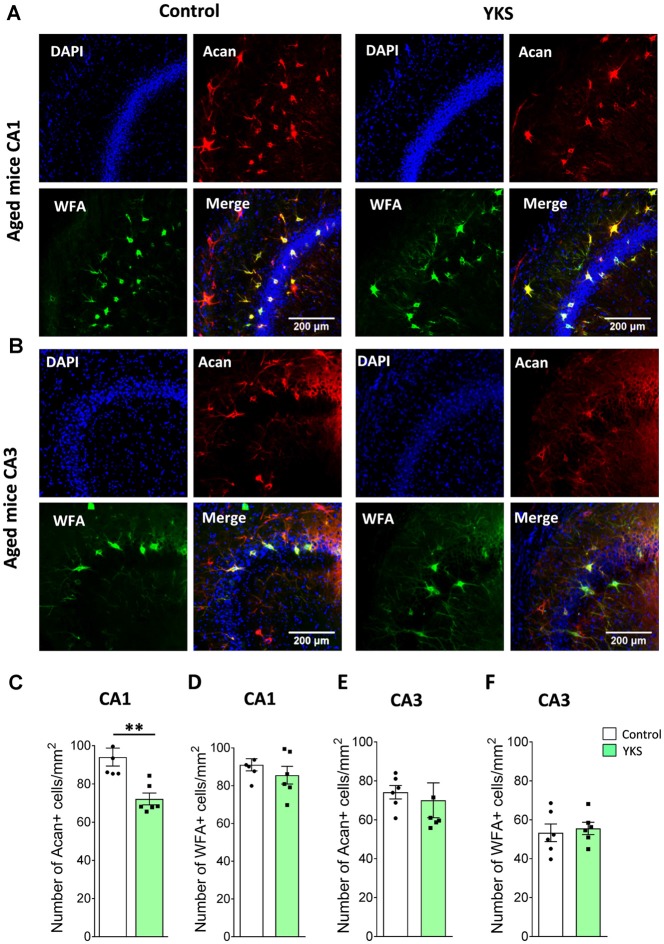
Effects of YKS on perineuronal nets (PNNs) in aged mice. **(A)** Representative images of PNNs stained by Aggrecan antibodies (red) and Wisteria Floribunda Agglutinin (WFA; green) in the CA1 (**A**) and CA3 (**B**) regions of the hippocampus of control and YKS-treated aged mice. Nuclei are stained with Hoechst stain (blue). Scale bar: 200 μm. Number of Aggrecan+ and WFA+ cells normalized per mm^2^ are shown for CA1 in **(C,D)** and CA3 in **(E,F)**. Bar graph represents the mean ± SEM values. **p < 0.01, Student’s *t*-test. *N* = 5 for control and *N* = 6 for YKS-treated aged mice for CA1 and *N* = 6 for both control and YKS-treated mice for CA3.

**Figure 3:**
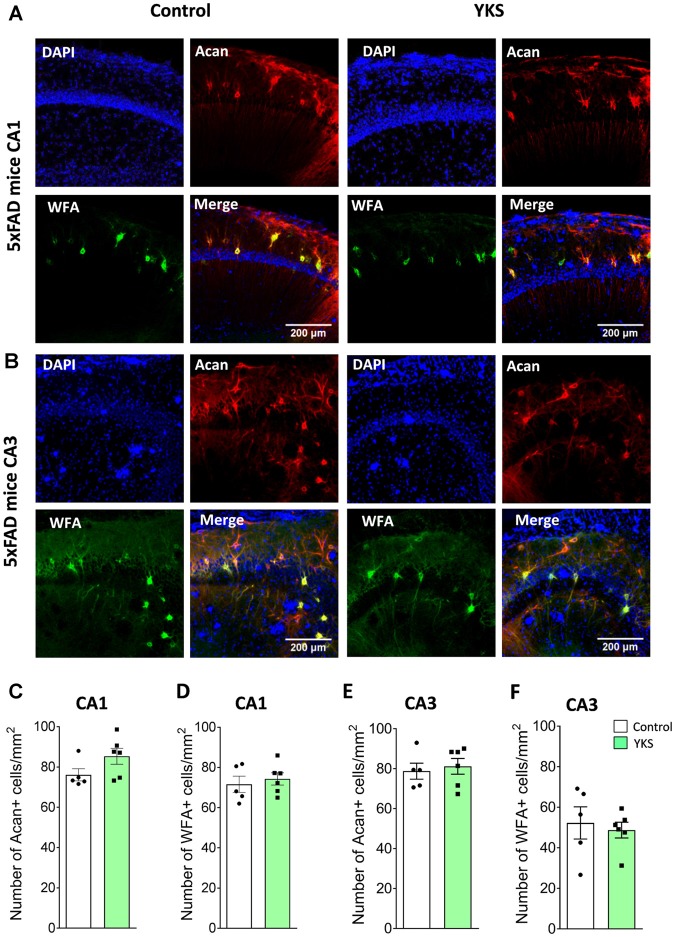
Effects of YKS on PNNs in 5xFAD mice. **(A)** Representative images of PNNs stained by anti-aggrecan antibodies (red) and biotinylated WFA (green) in the CA1 (**A**) and CA3 (**B**) regions of the hippocampus of control and YKS-treated 5xFAD mice. Nuclei are stained with Hoechst stain (blue). Scale bar 200 μm. Numbers of Aggrecan+ and WFA+ cells are shown for CA1 in **(C,D)** and for CA3 in **(E,F)**. Bar graphs represent the mean ± SEM values. There was no difference between YKS-treated and control groups by Student’s *t*-test. *N* = 5 for control mice for both CA1 and CA3 analysis and *N* = 6 for YKS-treated for both CA1 and CA3 analysis.

To evaluate the effects of YKS treatment on PNNs in aged and 5xFAD mice, we counted the numbers of Acan+ and WFA+ neurons in the hippocampal CA1 and CA3 areas. As previously reported for aged rats, we observed that YKS decreased the number of Acan+ cells by ≈20% (94.1 ± 4.7 vs. 72.2 ± 3.1; *t*-test, *p* = 0.003) in the CA1 area of the aged mice. Surprisingly, however, we did not observe any effect of YKS on the number of WFA+ neurons either in the CA1 or CA3 area in the aged mice (Figure 2). When such an analysis was performed with samples from the 5xFAD mice, no change was detected in the density of either Acan+ or WFA+ cells in the CA1 or CA3 areas of the hippocampus (Figure 3). These data suggest a potential difference in the molecular mechanisms involved in YKS effects on PNNs in aged and AD model mice.

### YKS Decreases Reactive Astrocytes in Aged Mice but Not in 5xFAD Mice

**Figure 4:**
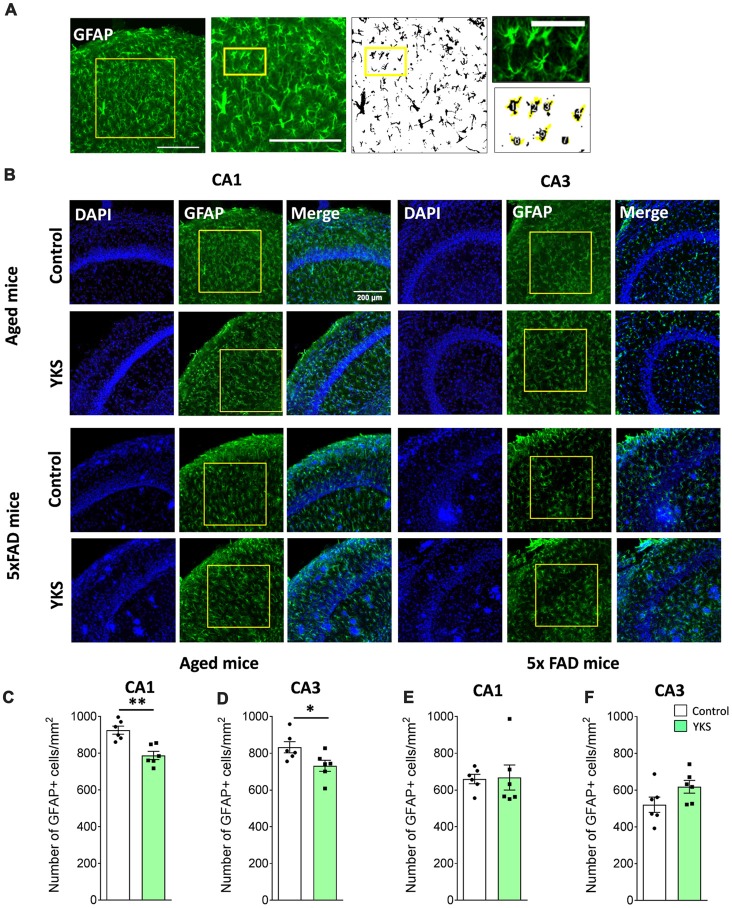
Effect of YKS on the reactive astrocytic population in aged and 5xFAD mice. **(A)** Scheme representing the method used for quantification of reactive glial cells using immunostaining for glial fibrillary acidic protein(GFAP). A square area spanning the hippocampal CA1 or CA3 area was selected and converted to a binary signal and automatically analyzed for counted particles in order to count the number of GFAP-positive cells using customized Fiji macros. Representative images of GFAP+ glial cells **(B)** and their respective quantification **(C–F)** in both the CA1 and CA3 areas of the hippocampus of control and YKS-treated aged mice as well as 5xFAD mice. Bar graphs represent the mean ± SEM values. Scale bars for **(A)** are 200 μm, 200 μm and 60 μm from left to right. *p < 0.05, **p < 0.01, Student’s *t*-test. *N* = 6 for all analyses.

To follow up on the mechanism that underlies the decrease in PNN after the YKS treatment, we investigated YKS effects on astroglial cells using GFAP as a marker for astrogliosis. Interestingly, YKS appeared to specifically decrease the number of GFAP+ cells in both the CA1 (925.8 ± 21.8 vs. 787.8 ± 22.3; *t*-test, *p* = 0.0013) and CA3 (832.7 ± 30.3 vs. 732.2 ± 30.1; *t*-test, *p* = 0.04) areas of the hippocampus in the aged mice (Figure 4). Similar to the results of the analysis of Acan+ cells, we could not detect any changes in the number of GFAP+ cells in the 5xFAD mice (Figure 4), suggesting that the specific decrease in PNNs following YKS treatment might be related to the decrease in activation of astrocytes.

### YKS Supports Mild Activation of Microglia in Aged Mice but Not in 5xFAD Mice

**Figure 5:**
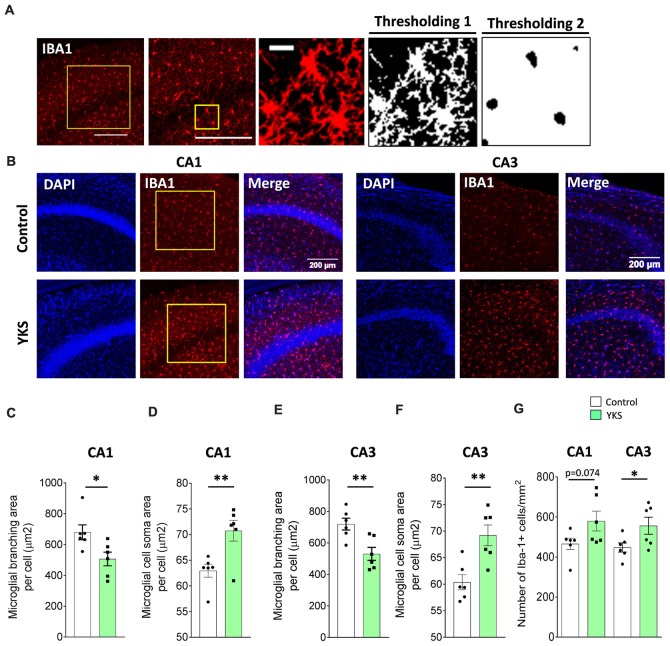
Effects of YKS on microglial activation in aged mice. **(A)** Scheme representing the method used for quantification of microglial activation using immunostaining for microglial marker ionized calcium-binding adaptor molecule 1 (IBA1). A square area spanning the hippocampal CA1 or CA3 area was selected and subjected to thresholding 1, converted to a binary signal and automatically analyzed to measure area and number of particles in order to calculate the average area covered by the branching of IBA1-positive cells. Additionally, the same image was subjected to different thresholding (Thresholding 2) in order to count the area covered by the soma of IBA1-positive cells. Representative images of IBA1 immunostaining **(B)** and the respective quantification of the area covered by microglial branching **(C,E)** and the area covered by microglial soma **(D,F)** in both the CA1 and CA3 areas of the hippocampus of control and YKS-treated aged mice. The number of IBA1-positive cells also seems to be increased in YKS-treated mice **(G)**. Bar graphs represent the mean ± SEM values. Scale bars for **(A)** are 200 μm, 200 μm and 20 μm from left to right. *p < 0.05, **p < 0.01, Student’s *t*-test. *N* = 6 for all analysis.

Next, we immunostained microglia using IBA1 antibodies in order to investigate the microglial activation (Figures 5A–G). Furthermore, using Fiji macros that were developed to measure the soma and the branching area of IBA1+ cells, we discovered that YKS treatment led to a decrease in the branching of microglial cells in both the CA1 (679.7 ± 48.4 vs. 506.7 ± 44.4 μm^2^ per cell; *t*-test, *p* = 0.0249) and CA3 (719.9 ± 37.6 vs. 531.2 ± 41.1 μm^2^ per cell; *t*-test, *p* = 0.0069) areas of the hippocampus in the aged mice. The decrease in branching of microglia was accompanied by a respective increase in the soma size in both the CA1 (62.9 ± 1.23 vs. 70.8 ± 2.03 μm^2^ per cell; Student’s *t*-test *p* = 0.008) and CA3 (60.4 ± 1.4 vs. 69.2 ± 1.9 μm^2^ per cell; Student’s *t*-test *p* = 0.0045). This analysis suggested that treatment with YKS leads to increased activation of microglia in both the CA1 and CA3 areas of the hippocampus in the aged mice. Furthermore, there was a significant increase in the number of microglia cells after YKS in CA3 (448.7 ± 23.0 vs. 556.5 ± 42.5 per mm^2^; *t*-test, *p* = 0.0486) as well as a similar tendency in CA1 (465.9 ± 28.7 vs. 579.7 ± 49.4 per mm^2^; *t*-test *p* = 0.074) areas of the hippocampus. A morphological analysis of microglial activation in the 5xFAD mice was impossible because of the extensive clustering of microglial cells around the amyloid plaques (Supplementary Figure S3). Thus, we measured the total intensity of IBA1 immunofluorescence per area of CA1 in the hippocampus. There was no difference in the intensity of IBA1 in the 5xFAD mice after YKS treatment (Supplementary Figure S3), which suggests no major changes in the activation of microglial cells following YKS treatment.

### YKS Does Not Affect the Aβ-Plaque Load

To investigate the effects of YKS on the Aβ plaques, we counted the number and area of Aβ plaques using DAPI and Aβ antibodies in the hippocampal CA1 area of the 5xFAD mice (Supplementary Figures S4A–C). We did not detect any effects of YKS either on the number or the area of plaques.

### YKS Might Impart Beneficial Effects via Enhancement of Cell Survival Signaling in Aged and 5xFAD Mice

**Figure 6:**
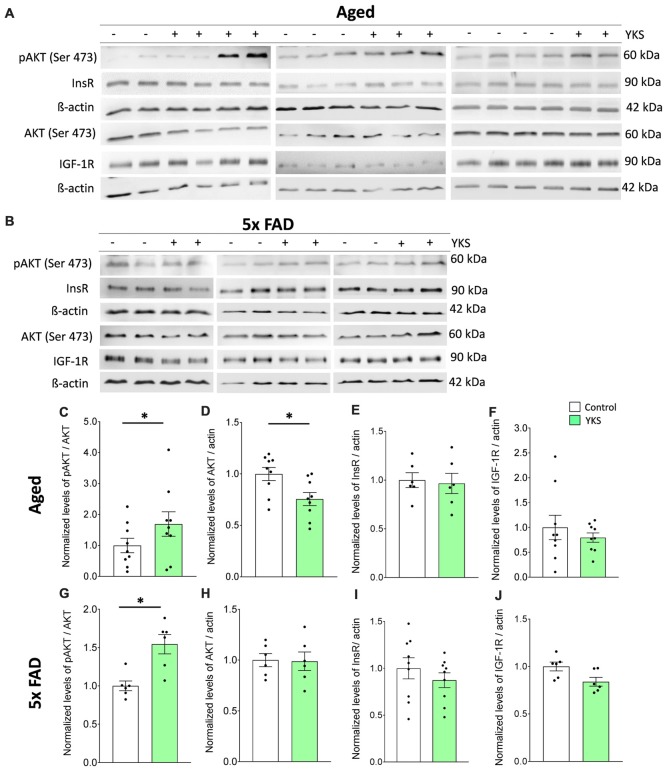
Effects of YKS on cell survival signaling. **(A,B)** Immunoblot analysis of total cortex homogenates from both aged as well as 5xFAD mice fed with (+) or without YKS (−). **(C,D)** Bar graph of phospho-Akt (pAkt) normalized to the total level of Akt and pan Akt normalized to the actin level for the aged mice. **(G,H)** Bar graph of pAkt normalized to the total level of Akt and pan Akt normalized to the actin level for the 5xFAD mice. **(E)** Bar graph of InsR normalized actin in aged mice. **(F)** Bar graph of IGF-1R normalized to actin level in aged mice. **(I)** Bar graph of InsR normalized to actin level in 5xFAD mice. **(J)** Bar graph of IGF-1R normalized to actin level in 5xFAD mice. Bar graphs represent the mean ± SEM values. *p < 0.05, Student’s *t*-test. *N* = 9 for aged animals and *N* = 6 for 5xFAD mice.

Impaired insulin (InsR) and insulin growth factor receptor (IGF-1R) signaling via pAkt/Akt has been linked to cognitive decline in normal aging, as well as AD (O’Neill, 2013; Akintola and van Heemst, 2015; Kullmann et al., 2016). Therefore, we examined the cortical tissue from both the aged and 5xFAD mice to investigate the effects of YKS on the levels of cell-protective pAkt, InsR and IGF-1R. We processed total homogenates of the cortices from the aged and 5xFAD mice and probed them following immunoblotting with anti-pan-Akt or pAkt antibodies (Figures 6A–D,G,H). We identified an increase in the ratio of pAkt/Akt after treatment with YKS in the aged mice (1.0 ± 0.23 vs. 1.7 ± 0.39; *t*-test, *p* = 0.04) and the 5xFAD mice (1.0 ± 0.06 vs. 1.54 ± 0.12; *t-test*, *p* = 0.02), indicating enhanced Akt activity. Interestingly, we also observed a significant decrease in the total levels of Akt in the YKS-treated aged mice compared to the control animals (1.0 ± 0.06 vs. 0.75 ± 0.06; *t*-test, *p* = 0.02). Notably, the InsR and IGF-1R levels were not altered by the YKS treatment, which suggests that Akt activity is regulated via upstream mechanisms independent of InsR and IGF-1R (Figures 6A,B,E,F,I,J).

## Discussion

In the present study, we systematically compared the therapeutic potential of the herbal Kampo medicine YKS in two distinct groups of animals: healthy aged mice and the 5xFAD model of early-onset familial AD. To this end, we evaluated YKS mediated behavior and the associated cellular and molecular changes in the hippocampus and cortex of these animals. Our data suggest YKS mediates vastly different cellular and molecular responses in healthy aging brains in comparison to 5xFAD mouse model of AD, as summarized in Table 2. Interestingly, we identified marked improvements in the cognitive abilities of both the aged and 5xFAD mice following YKS treatment, suggesting a beneficial role of YKS in both conditions. Cognitive improvement in the aged mice was associated with reduced expression of the ECM of PNNs, reduced astrogliosis, increased microglial activation, and an increased neuroprotective pAkt/Akt ratio. In the 5xFAD mice, we did not identify YKS-mediated effects on the ECM, astrogliosis and microglial activation. However, we identified an increase in neuroprotective pAkt/Akt signaling similar to that in the aged mice, thus dissecting a common mechanism by which aged and 5xFAD brains respond to YKS.

**Table 2:** Summary of YKS effects.

Parameter	Aged mice	5xFAD mice
Novel object recognition	Improved	Improved
Contextual fear conditioning	Improved	Improved
Working memory	=	=
Extracellular matrix (PNN)	decreased	=
Astrogliosis	decreased	=
Microglia activation	Increased	=
Neuroprotective pAkt/Akt	Increased	Increased
InsR	=	=
IGF1-R1	=	=
Aβ plaques	=	=

*=, not changed*.

Most clinical trials with YKS have shown selective improvements in BPSD symptoms, without significant improvements in cognitive abilities as measured by the Mini Mental State Examination (Iwasaki et al., 2005; Matsuda et al., 2013; Mizoguchi and Ikarashi, 2017a, b). A recent randomized, double-blind, placebo-controlled clinical trial in AD patients also revealed some selective improvements in BPSD symptoms and no impact on cognitive tests (Furukawa et al., 2017). In contrast, animal studies have shown that YKS treatment results in cognitive improvement (Uchida et al., 2013). Similarly, we found a significant increase in the DR during the NORT and CFC, reflecting improvements in hippocampal functioning. The differences in the outcomes of the present study from those of human studies may be related to the fact that the 5xFAD mouse corresponds to the early familial form of AD, whereas the clinical trials were conducted on patients with the sporadic form of AD who had a mean age of 80 ± 9 years (Iwasaki et al., 2005). The mechanisms that underlie the sporadic form of AD might involve an interaction between age-associated gradual changes and amyloidosis, which was not modeled in the middle-aged 5xFAD mice used in our study. In addition, the duration of the treatments with YKS in humans was shorter (1 month, 0.1% of an 80-year lifespan) than the duration used in this study (2 months plus the time of behavioral assessment, at least 8.3% of a 2-year lifespan; Iwasaki et al., 2005; Mizukami et al., 2009). Based on our results, we speculate that YKS might be beneficial for cognition in the early familial form of AD or in cases of sporadic AD if administered for longer periods of time and potentially at earlier stages of the disease.

The neural ECM is a meshwork of various molecules, which fills the extracellular space between neurons, glia and vascular elements, together with the interstitial fluid. It is composed of hyaluronic acid as the backbone to which chondroitin sulfate proteoglycans and link proteins bind and are further stabilized by tenascins (Dityatev et al., 2010; Sorg et al., 2016). Matrix molecules are not only present in the interstitial space but are arranged into highly dense structures, such as PNNs, around the soma and proximal neurites of parvalbumin-containing interneurons in many brain areas, such as the hippocampus and cortex (Dityatev et al., 2007). Previous studies have shown that YKS decreases the expression of the PNN-forming matrix protein aggrecan in aged and young rats (Tanaka and Mizoguchi, 2009). We also identified similar, although less dramatic, changes in PNNs following the treatment of aged mice with YKS. The smaller YKS effect in our study might be related to the facts that: (1) aggrecan analysis was performed after behavioral experiments rather than in naïve mice, as was done previously; and (2) learning may induce remodeling of the ECM (Favuzzi et al., 2017). Surprisingly, YKS treatment led to a decrease in aggrecan expression but not in WFA labeling. In adult mice, WFA almost exclusively correlates with aggrecan (Costa et al., 2007); however, in aged mice, the carriers of WFA-binding glycan have not previously been characterized. It is possible that the YKS-mediated decrease in the expression of aggrecan was compensated by increased expression of other lecticans, such as brevican, or by changes in the expression/availability of glycoepitopes detected by WFA (Ueno et al., 2017). Another novel observation in this study is that YKS did not decrease the expression of ECM in the 5xFAD mice, which suggests that the mechanisms of YKS action in aged and AD mice are distinct.

In light of the accumulating evidence on the beneficial effects of an enriched environment and cognitive and physical exercise on brain neurogenesis, the vascular system and ECM remodeling, it is highly tempting to test the synergistic effects of YKS in combination with an enriched environment in 5xFAD and other dementia models (Madinier et al., 2014; Johansen-Berg and Duzel, 2016). As YKS improves behavior without affecting the expression of amyloid plaques, it would be of substantial interest to investigate YKS as a supplementary treatment in combination with Aβ clearance immunotherapy.

Other researchers have intensively investigated YKS-mediated pharmacological mechanisms against glutamate-mediated excitotoxicity (Ikarashi et al., 2009). It has been shown that YKS leads to an increase in glutamate uptake by astrocytes, thereby decreasing neuronal death in a dose-dependent manner (Kawakami et al., 2009). In the context of our study, it is noteworthy that there is an age-dependent increase in astroglial activation (Jiang and Cadenas, 2014), which correlates with reduced expression of glutamate transporter 1 (GLT1), thus suggesting an impaired function of glutamate clearance in aging (Xin et al., 2009). Reactive astrogliosis may also lead to increased expression of inhibitory ECM molecules (Wiese et al., 2012). Our data regarding a reduction in GFAP+ astrocytes after YKS treatment support the notion that YKS ameliorates astrogliosis in aged mice. Changes in astrocytic state might lead not only to increased scavenging of glutamate (and may thus be neuroprotective) but also to a reduction in ECM expression that might, in turn, support cognitive flexibility, brain plasticity and cognition (Morellini et al., 2010; Happel et al., 2014; Végh et al., 2014b).

Recently, YKS has been shown to ameliorate neuronal cell death mediated by cerebral ischemia in gerbils by potentially mitigating the microglial inflammatory response (Liu et al., 2014). Moreover, YKS reduces the intensity of CD11+ immunoreactivity, promotes neurogenesis and ameliorates cognitive deficits in Gunn rats used as a model of schizophrenia (Furuya et al., 2013). However, in both studies, the conclusion was based on the analysis of the number and intensity of IBA1+ and CD11b+ cells. IBA1 and CD11b are general markers of microglia and macrophages and are not specific markers of microglial activation (Matsumoto et al., 2007; Han et al., 2014). Microglial activation is a complex process that follows various stages of morphological changes, which start from the physiological ramified nonreactive state to the activated amoeboid state. Thus, morphology-based analysis becomes increasingly important to measure their activation. Recent evidence suggests that mild microglial activation might be neuroprotective; however, strong microglial activation, such as that induced by brain injury, exacerbates brain damage and triggers morphological changes in microglia from ramified to amoeboid (Streit, 2002; Marín-Teva et al., 2004; Müller et al., 2006; Simard et al., 2006; Ziv et al., 2006; Block et al., 2007). In our study, for the first time, we identified a highly significant increase in the soma size and a corresponding decrease in the branching area of microglia after YKS treatment, with the gross morphology being ramified rather than amoeboid. Thus, our data suggest a modest increase in the activation of microglia, which might support physiological neuroprotective functions, as a new function of YKS in aged brains.

Neuroinflammation is characterized by increased activation of both microglia and astroglia (Sofroniew and Vinters, 2010; Heppner et al., 2015). Several studies have shown that activation of both microglia and astrocytes may be both neuroprotective and neurodegenerative (Martinez and Gordon, 2014; Sofroniew, 2014; Anderson et al., 2016). Recently, it has been shown in a landmark study that astrocytic activation may be further characterized by two different activation states of astrocytes referred to as A1 and A2. The A1 type of reactive astrocytes might be harmful to neurons, whereas A2 is neuroprotective (Liddelow et al., 2017). Furthermore, the authors show that increased activation of microglia following LPS stimulation transformed astrocytes into the A1 state, whereas middle cerebral artery occlusion to induce ischemia transformed them into the A2 state, which suggests that different types of brain insults might induce opposite types of microglial and astrocytic activation. Interestingly, we showed that YKS decreases astrocytic activation while increasing microglial activation. However, we did not further dissect the type of microglial and astroglial activation. Considering the strong behavioral benefits of YKS in both aged and AD animals, it is possible that both the microglial activation and decreased astrocytic activation by YKS might be neuroprotective. However, an astrocytic and microglial specific gene expression profiling or cytokine profiling should be performed in the future to obtain additional insights into glial regulation by YKS.

There is mounting evidence that AD is closely linked to dysregulated serotonin (5-HT) receptor signaling and insulin resistance in the brain (Kepe et al., 2006; de la Monte et al., 2006; Cirrito et al., 2011; Xu et al., 2012). Brain insulin signaling-related molecules, such as IGF-IR and InsR, are upstream mediators of neuroprotective PI3K/Akt/GSK-3β signaling and are dysregulated in the AD brain (Bondy and Cheng, 2004; Lee et al., 2005; Messier and Teutenberg, 2005; Moloney et al., 2010). Moreover, signaling through neuronal PI3-kinase/Akt/mTOR pathway or its activation by insulin and IGF-1 has been shown to be protective against the development of AD pathology (Oddo, 2012; Cai et al., 2015). Our results for the first time demonstrate *in vivo* that YKS might mediate neuroprotection against Aβ-mediated neuronal toxicity via increased signaling through pAkt. This finding is in line with previous *in vitro* studies implicating an increase in Akt signaling as a YKS-mediated neuroprotective mechanism. Surprisingly, we could not identify direct changes in the levels of InsR or IGF-1R, suggesting that YKS acts on pAkt pathway independent of insulin signaling. It is noteworthy here that YKS is composed of several active components, including geissoschizine methyl ether and hirsuteine (Mizoguchi and Ikarashi, 2017a, b). Both compounds have been implicated as potent agonists of 5-HT1A receptors (Kanno et al., 2013; Kushida et al., 2015) and detected in the plasma and the brain after the oral administration of YKS, suggesting a potential activation of 5-HT1A receptor signaling by YKS (Kitagawa et al., 2015). Importantly, in hippocampal cultured neurons, an increased signaling via 5-HT1 receptors is coupled to increased activation of Akt (Cowen et al., 2005), suggesting that YKS mediated activation of Akt pathway might be due to increased activation of 5-HT1A receptor signaling, but alternative/additional mechanisms converging on activation of Akt cannot be excluded.

In summary, our study supports the view that prolonged YKS treatment might be beneficial for cognitive improvements in aging and the early-onset familial form of AD and outlines the mechanisms involved. An important continuation of this work would be to dissect the pAkt-mediated up- and downstream signaling pathways and to perform high-throughput *in vitro* analysis of astrogliosis, microglia activation and Akt activation after fractionation of YKS to isolate its active components. YKS can then be combined with emerging treatments that target other aspects of AD that are not efficiently targeted by YKS, such as Aβ clearance immunotherapy to reduce the Aβ load, physical and cognitive brain training to improve vascularization, and/or the downregulation of neural ECM expression. This line of research might eventually provide safe and more potent “combinatorial” treatments for dementia.

## Author Contributions

RK, EM, OS and AD designed the research study and wrote the manuscript. RK and JS performed immunohistochemical experiments for ECM, astrocytic activation, microglial activation and Aβ. RK analyzed these data. EM performed the behavioral experiments and behavioral data was analyzed by EM and OS. AC performed Western blotting for pAkt, InsR and IGF-1R and analyzed the data. All authors actively reviewed and edited the manuscript. All authors read and approved the final manuscript.

## Conflict of Interest Statement

The authors declare that the research was conducted in the absence of any commercial or financial relationships that could be construed as a potential conflict of interest.
