# Supplementary material for: Traditional Japanese Herbal Medicine Yokukansan Targets Distinct but Overlapping Mechanisms in Aged Mice and in the 5xFAD Mouse Model of Alzheimer’s Disease
Source: Front Aging Neurosci. 2018 Dec 17;10:411. doi: 10.3389/fnagi.2018.00411 (PMC6315162; doi:10.3389/fnagi.2018.00411)
Supplement: Supplementary file 1 [file Presentation_1.pptx]

## Slide 1
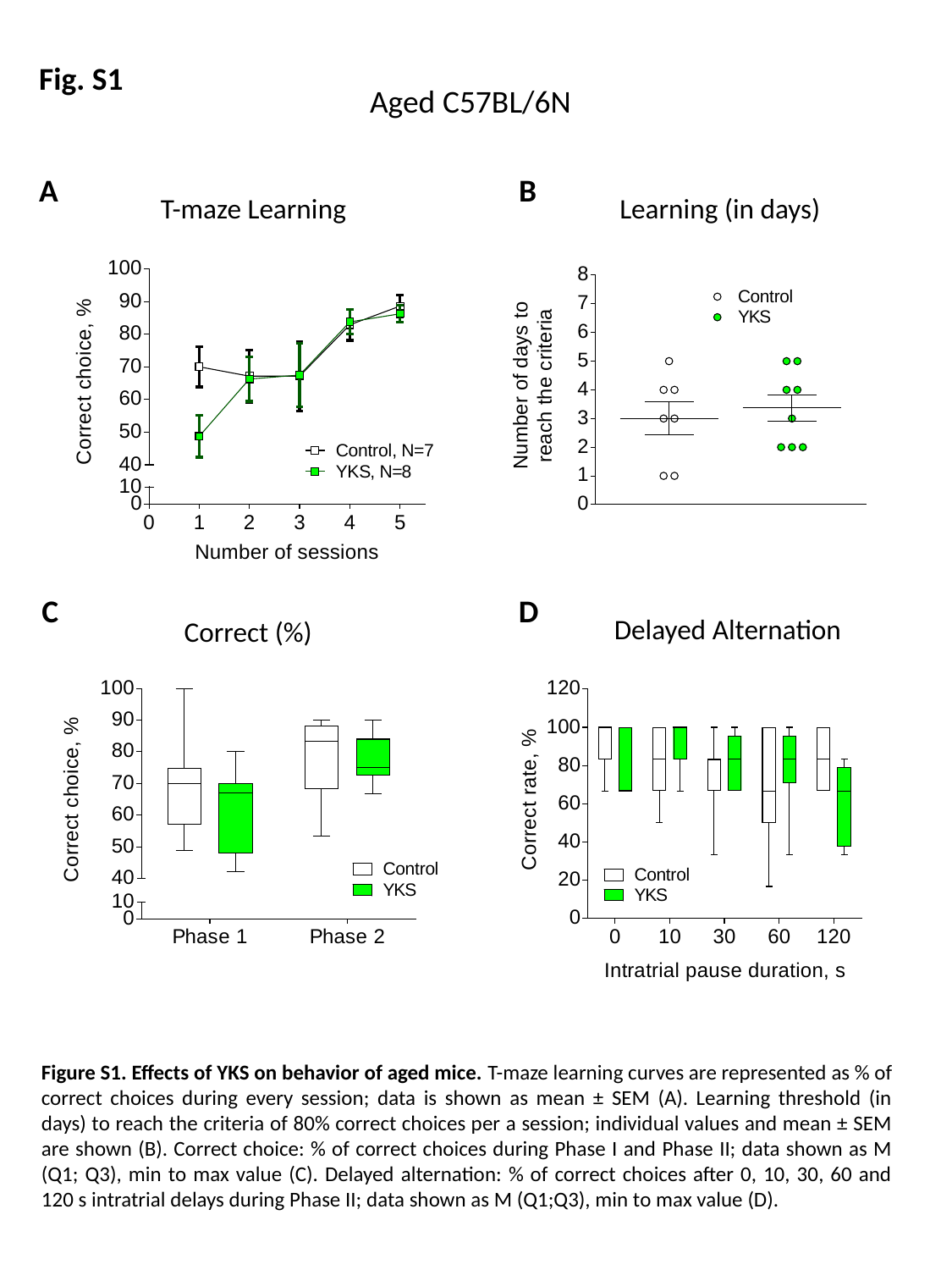

Fig. S1
Aged C57BL/6N
A
B
T-maze Learning
Learning (in days)
C
D
Delayed Alternation
Correct (%)
Figure S1. Effects of YKS on behavior of aged mice. T-maze learning curves are represented as % of correct choices during every session; data is shown as mean ± SEM (A). Learning threshold (in days) to reach the criteria of 80% correct choices per a session; individual values and mean ± SEM are shown (B). Correct choice: % of correct choices during Phase I and Phase II; data shown as M (Q1; Q3), min to max value (C). Delayed alternation: % of correct choices after 0, 10, 30, 60 and 120 s intratrial delays during Phase II; data shown as M (Q1;Q3), min to max value (D).

## Slide 2
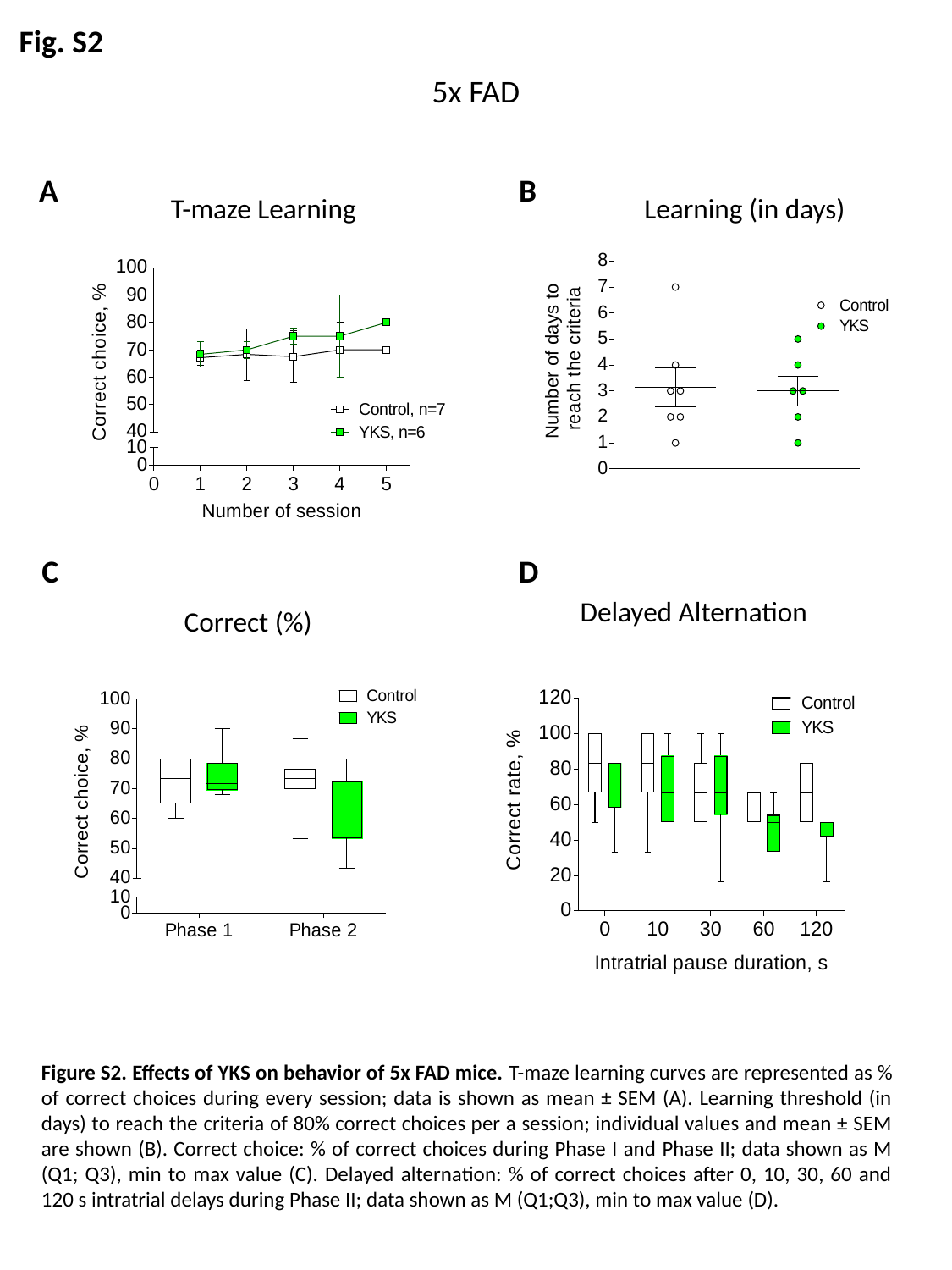

Fig. S2
5x FAD
A
B
T-maze Learning
Learning (in days)
C
D
Delayed Alternation
Correct (%)
Figure S2. Effects of YKS on behavior of 5x FAD mice. T-maze learning curves are represented as % of correct choices during every session; data is shown as mean ± SEM (A). Learning threshold (in days) to reach the criteria of 80% correct choices per a session; individual values and mean ± SEM are shown (B). Correct choice: % of correct choices during Phase I and Phase II; data shown as M (Q1; Q3), min to max value (C). Delayed alternation: % of correct choices after 0, 10, 30, 60 and 120 s intratrial delays during Phase II; data shown as M (Q1;Q3), min to max value (D).

## Slide 3
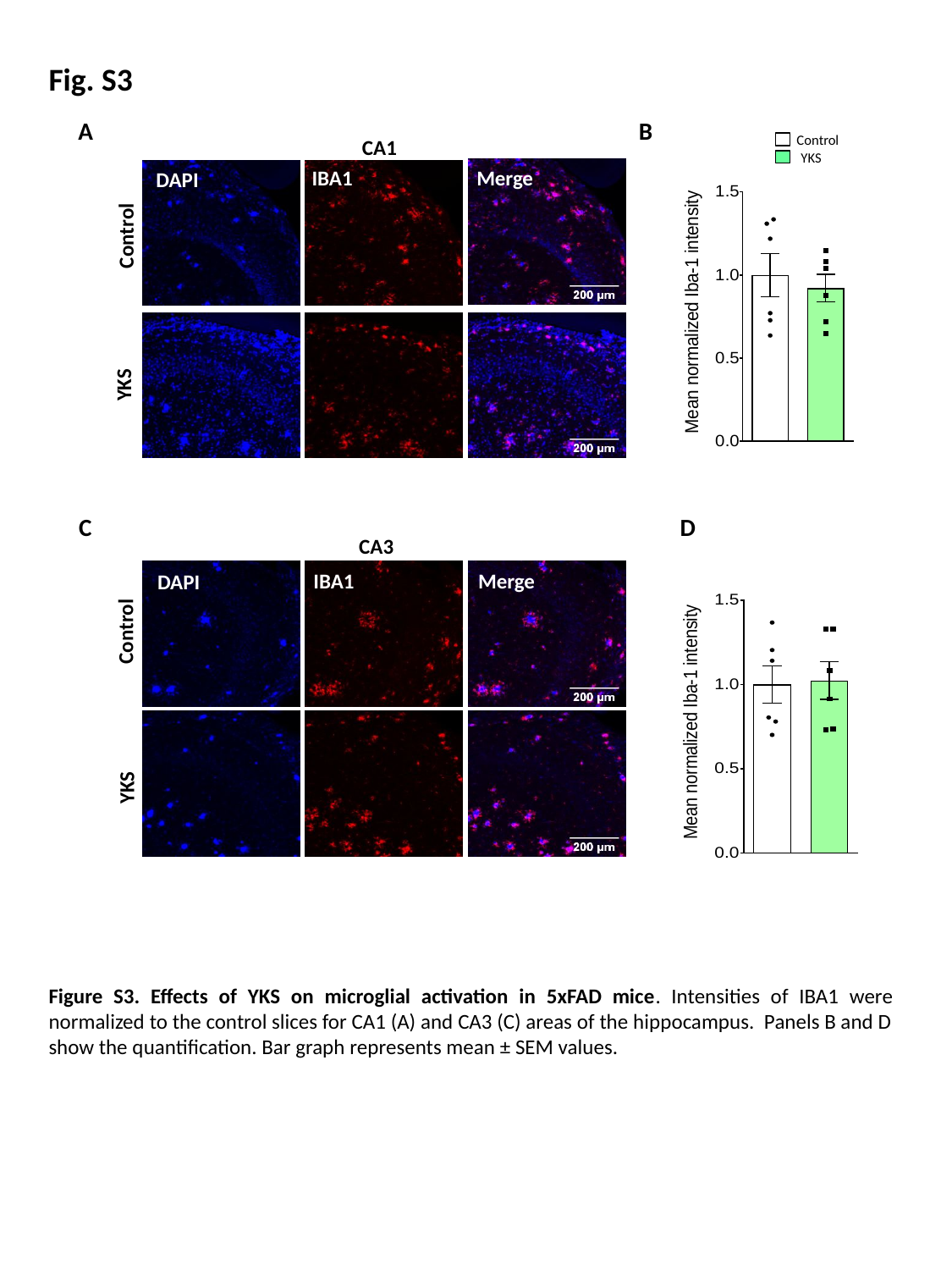

Fig. S3
A
B
Control
YKS
CA1
Merge
IBA1
DAPI
Control
YKS
C
D
CA3
Merge
IBA1
DAPI
Control
YKS
Figure S3. Effects of YKS on microglial activation in 5xFAD mice. Intensities of IBA1 were normalized to the control slices for CA1 (A) and CA3 (C) areas of the hippocampus. Panels B and D show the quantification. Bar graph represents mean ± SEM values.

## Slide 4
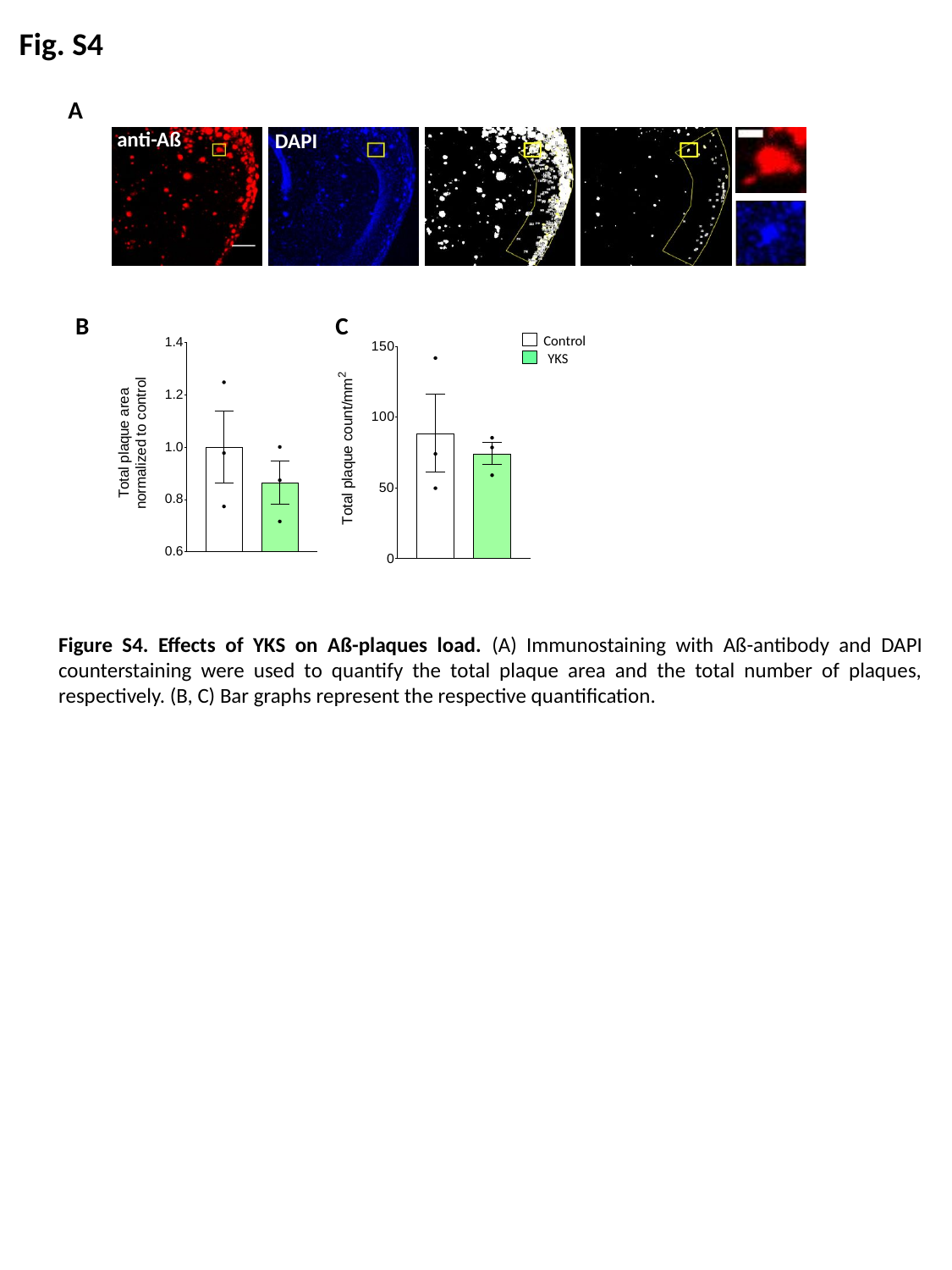

Fig. S4
A
anti-Aß
DAPI
B
C
Control
YKS
Figure S4. Effects of YKS on Aß-plaques load. (A) Immunostaining with Aß-antibody and DAPI counterstaining were used to quantify the total plaque area and the total number of plaques, respectively. (B, C) Bar graphs represent the respective quantification.
